# Synergistic Anti-Angiogenic Effect of Combined VEGFR Kinase Inhibitors, Lenvatinib, and Regorafenib: A Therapeutic Potential for Breast Cancer

**DOI:** 10.3390/ijms23084408

**Published:** 2022-04-16

**Authors:** Khuloud Bajbouj, Rizwan Qaisar, Mohammed A. Alshura, Zeinab Ibrahim, Mohamad B. Alebaji, Amenah W. Al Ani, Hanadi M. Janajrah, Mariah M. Bilalaga, Abdelrahman I. Omara, Rebal S. Abou Assaleh, Maha M. Saber-Ayad, Adel B. Elmoselhi

**Affiliations:** College of Medicine, University of Sharjah, Sharjah P.O. Box 27272, United Arab Emirates; kbajbouj@sharjah.ac.ae (K.B.); rqaisar@sharjah.ac.ae (R.Q.); mhd97sh@gmail.com (M.A.A.); zainab-m-ibrahim@hotmail.com (Z.I.); mohamadbaraa1@hotmail.com (M.B.A.); amna-alani96@hotmail.com (A.W.A.A.); hanadi969@yahoo.com (H.M.J.); mariahbilalaga@gmail.com (M.M.B.); abdelrahman.ib.omara@gmail.com (A.I.O.); u16103002@sharjah.ac.ae (R.S.A.A.); msaber@sharjah.ac.ae (M.M.S.-A.)

**Keywords:** breast cancer, VEGFR kinase inhibitors, Lenvatinib, Regorafenib, angiogenesis, cancer therapy, chemotherapy

## Abstract

Background: Breast cancer currently affects more than two million women worldwide, and its incidence is steadily increasing. One of the most essential factors of invasion and metastasis of breast cancer cells is angiogenesis and non-angiogenic vascularization. Lenvatinib and Regorafenib share the same anti-angiogenic effect by inhibiting vascular endothelial growth factor receptors (VEGFRs subtypes 1 to 3) and have been approved for treating different types of cancer. Methods: We investigated Lenvatinib and Regorafenib effects on a well-established in-vitro model of breast cancer using MCF-7 (estrogen, progesterone receptor-positive, and HER2-negative), MDA-MB-231 (triple negative), as well as Human Umbilical Vascular Endothelial Cell line (HUVEC) cell lines. We performed the cell viability assay on four groups of cells, which included a control group, a Lenvatinib treated only group, a Regorafenib treated only group, and a group treated with a combination of both drugs at 24, 48, and 72 h. Data were analyzed as means ± standard deviation, and the drug–drug interactions with Compusyn software. Cellular migration assay, tube formation assay, and Western blots were conducted to determine the functional and the protein expression of downstream signals such as Caspase-9, anti-apoptotic Survivin, P-ERK, and total-ERK in the control and treatment groups. Results: MCF-7 cells showed a reduction in cell survival rates with higher dosing and longer incubation periods with each drug and with the combination of drugs. A synergistic interaction was identified (CI < 1) with both drugs on MCF7 at different dose combinations and at a higher dose in MDA-MB-231 cells. Furthermore, there was a marked decrease in the anti-angiogenic effect of both drugs in tube formation assay using MDA-MB-231 cells and survivin protein expression in MCF-7, and those antitumor markers showed a better outcome in drug combination than the use of each drug alone. Conclusion: Our result is the first to report the synergistic anti-angiogenic potential of combination therapy of Lenvatinib and Regorafenib. Therefore, it shows their therapeutic potential in breast cancer, including the aggressive types. Further studies are warranted to confirm and explore this therapeutic approach.

## 1. Introduction

Breast cancer is the most common cancer in women, with a higher mortality rate than other cancers. It affected 2.3 million women worldwide in 2020, and the incidence is steadily increasing globally [1]. As the leading cause of cancer-related deaths in women, breast cancer requires rigorous and continuous characterization at the molecular level to further discover newer and more robust therapeutic targets. Several studies have reported the therapeutic potential of various drugs such as 5-fluorouracil, Regorafenib, and Lenvatinib using in-vitro models [2]. Among different breast cancer cell lines, MCF-7 and MDA-MB-231 cells, both originally derived from a patient with metastatic breast cancer, have emerged as useful in-vitro models of breast cancer and have been extensively used in research laboratories over the past forty years [3]. 

One of the most critical factors for the invasion and metastasis of breast cancer is angiogenesis and non-angiogenic vascularization [4,5]. Angiogenesis has been characterized as the formation of new blood vessels from pre-existing ones by either sprouting or intussusception [6]. On the other hand, non-angiogenic tumor cells grow in absence of new vessel formation [7]. Vascularization by non-angiogenic mechanisms includes vascular mimicry and vascular co-option. Vascular mimicry was described by Maniotis et al. [8] as a process by which cancer cells form microvascular channels de novo, while in vascular co-option the cancer cells invade and take over pre-existing blood vessels from non-tumoral surrounding tissue [9]. Vascular mimicry is primarily seen in uveal melanoma but has since also been observed in carcinomas of the prostate, kidney, bladder, lung, and breast. The importance of vascular mimicry lies in its association with high tumor grade metastasis and high patient mortality [7]. The other non-angiogenic mechanism, which is vessel co-option is a major mechanism of breast cancer cells metastasis to highly vascular organs including the liver, brain, and lungs. Vessel co-option also aids in breast cancer metastasis to the lymph nodes. This might be the reason behind the poor therapeutic efficacy of anti-angiogenic medications in breast cancer [10]. The exact mechanisms for vascular mimicry and co-option are not been completely unveiled; however, they share similar triggers and signaling pathways with angiogenesis such as hypoxia, VEGF-A among others. A thorough understanding of these molecular pathways in all processes of vascular formation is critical to characterizing the grades of neoplastic growth, identifying therapeutic targets, and developing new candidate drugs. Mitigating tumor angiogenesis and other vascularization processes can potentially reduce the proliferative potential of malignant neoplasms since cancerous masses require a sustained supply of oxygen and nutrients for their growth. However, even though the presence of vessels is essential for tumor growth and spread, the angiogenic ability of neoplastic cells, the quantity of the vessels, and the quality of the new vessels remain variable [11].

The vascular endothelial growth factor (VEGF) family is an essential driver of neo-vessels formation. Among its members, VEGF-A is the master regulator of physiological and pathological pathways controlling angiogenesis. Additionally, it has the ability to control and stimulate the migration of macrophages and endothelial cells, as well as the activity of vascular permeability. VEGF-A regulates angiogenesis by activating two tyrosine kinase receptors, namely VEGFR-1 and VEGFR-2. Due to its important role in angiogenesis, VEGF-A also emerged as an attractive candidate for targeted chemotherapy drugs. Other members of the VEGF family are VEGF-C, VEGF-D, and their receptor VEGFR-3, which is specifically responsible for the regulation of lymphangiogenesis [12]. MCF-7 cells express four different vascular endothelial growth factors: VEGF-A; VEGF-B; VEGF-C; and VEGF-D [13]. They also express VEGF receptor 1 [13]. The MCF-7 cell line is dependent on angiogenesis for its growth and progression, and any reduction in the expression of VEGF or VEGFR1 might be a potential therapeutic strategy to mitigate cancer cell growth.

Lenvatinib, approved by the FDA in 2018, is an oral, multi-targeted tyrosine kinase inhibitor (TKI) that explicitly suppresses the vascular endothelial growth factor receptor 1–3 (VEGFR1–3), the fibroblast growth factor receptor gene 1–4 (FGFR 1–4), the platelet-derived growth factor receptors A (PDGFR α), RET and KIT 3, 7. Clinical studies have determined that Lenvatinib blocks VEGF- and FGF-driven angiogenesis, KIT-dependent angiogenesis, RET-fusion/RET freak tumorigenesis, and VEGFR3-associated lymphangiogenesis in preclinical human thyroid cancer models [13]. Lenvatinib has also been utilized to treat progressive and metastatic refractory and unresectable thyroid malignancies [14]. It is also used in conjunction with Everolimus, an inhibitor of the mammalian target of rapamycin (mTOR), for the treatment of metastatic renal cell carcinoma by acting on the VEGF-targeted treatment [15].

Regorafenib (FDA-approved in 2012) is an oral multi-kinase inhibitor that targets angiogenic, stromal, and oncogenic kinases, including VEGF receptors 1, 2, and 3, epidermal growth factor homology domain 2 (TIE-2), platelet-derived growth factor receptor-β, c-kit, ret, raf-1, and BRAF in both wild-type and the V600E mutant colorectal cancer (CRC) cells in humans. In preclinical studies, Regorafenib demonstrates antitumor activity in a wide-ranging spectrum of preclinical models, as well as CRC in a first-in-human, non-randomized study [16]. In a phase II study, Regorafenib treatment resulted in a 31% partial response (PR) rate and 50% stabilization rate in patients with metastatic renal cell carcinoma. Regorafenib also inhibits both VEGFR-2 and TIE2 TK and may have an advantage over inhibition of the VEGF axis alone. A promising clinical outcome was shown in a phase I study in 27 patients with advanced refractory CRC with a 74% overall disease control rate [17].

In the current study, we investigated the combined effect of Regorafenib and Lenvatinib, using a well-established in-vitro of breast cancer in MCF-7 and MDA-MB-231 cell lines. The main goal is to explore if this combined therapeutic approach could be a potential alternative or adjunctive to the current therapies to reduce cancer growth and metastasis.

## 2. Results and Figures

**Figure 1:**
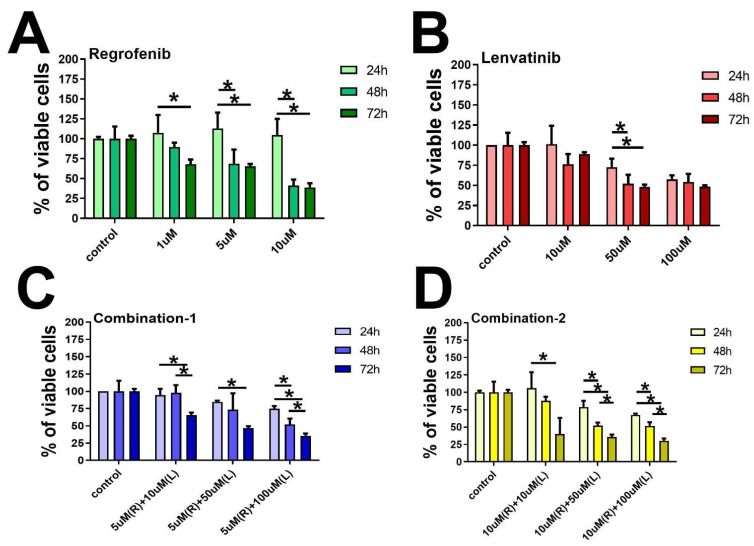
Effect of (**A**) Regorafenib (1, 5, and 10 µM), (**B**) Lenvatinib (10, 50, and 100 µM), (**C**) Regorafenib–Lenvatinib (Combination-1) = 5 μM Regorafenib + (10, 50, or 100) μM Lenvatinib, and (**D**) Regorafenib-Lenvatinib (Combination-2) = 10 μM Regorafenib + (10, 50, or 100) μM Lenvatinib, on cancer cell survival. MCF-7 cells were treated with various concentrations of Regorafenib, Lenvatinib, and a combination of both drugs. Dose and time-dependent survival of the cells were measured over a 24 to 72 h period using the MTT assay. * statistically significant from the control cells, *p*-value < 0.05.

**Figure 2:**
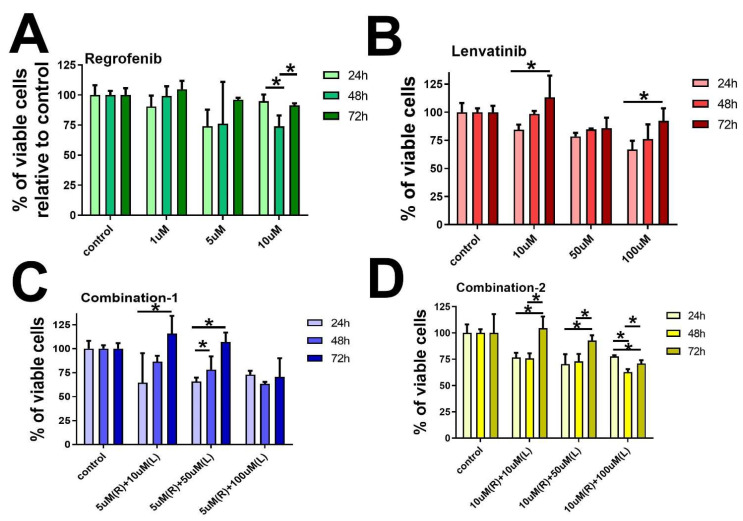
Effect of (**A**) Regorafenib (1, 5, and 10 µM), (**B**) Lenvatinib (10, 50, and 100µM), (**C**) Regorafenib–Lenvatinib (Combination-1) = 51 μM Regorafenib + (10, 50, or 100) μM Lenvatinib, and (**D**) Regorafenib–Lenvatinib (Combination-2) = 10 μM Regorafenib + (10, 50, or 100) 10 μM Lenvatinibon MD-MB-231 cell survival. MD-MBA-231 cells were treated with various concentrations of Regorafenib, Lenvatinib, and a combination of both drugs. Dose and time-dependent survival of the cells were measured over a 24 to 72 h period using the MTT assay. * statistically significant from the control cells, *p*-value < 0.05.

We found significantly reduced cell survival in the MCF-7 cells with Regorafenib in a time- and dose-dependent manner. The effects were less robust with Lenvatinib but more prominent with several combinations of the two chemotherapies (Figure 1). On the other hand, we found less reduction in the cell survivals in the MD-MB-231 cells with either of the two drugs, alone or in combination when compared to MCF-7 cells. However, there was a trend among all groups toward a reduction in cell survival with increased doses (Figure 2). It is important to note that the cell survival rate was high in the group treated with Regorafenib or Lenvatinib alone, in comparison with the group treated with both agents together, perhaps due to initial drug resistance.

There was a synergistic interaction between both compounds on MCF7 cells at different dose combinations (expressed by CI < 1), (Supplementary Materials). On the MDA-MB-231 (triple negative) cell line, synergism was observed only at a combination of high doses of 100 µM of Lenvatinib, (Supplementary Materials). At 72 h, the IC_50_ of Regorafenib was 7.12 µM on MCF7 and 4.36 µM on the MD-MB-231 cells. At 72 h, the IC_50_ of Lenvatinib was 12.12 µM on MCF7 and 11.27 µM on MD-MB-231 cells.

**Figure 3:**
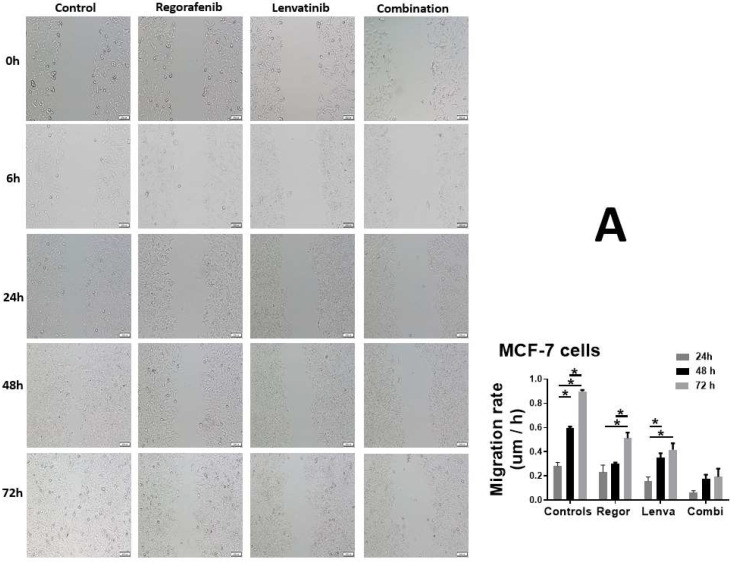

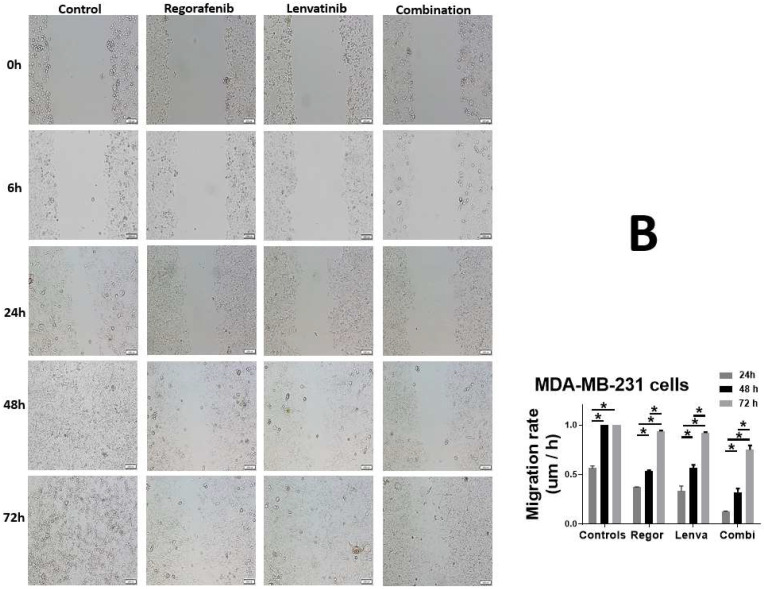
Effect of Regorafenib and Lenvatinib on cell migration. A scratch test was used to assess (**A**) MCF-7 and (**B**) MDMBA cell migration, and the width of the scratch was noted at the pre-defined time points of 6, 24, and 48 h. The scale bar corresponds to 100 µm for all panels. The untreated control cells showed significant cellular migration, while treatment with Regorafenib and Lenvatinib, either alone or in combination, prevented the migration of MCF-7 and MDMBA cells, confirming the anti-metastatic potential of these drugs. * Statistically significant from the control cells, *p*-value < 0.05.

We next performed the cellular migration assay on four groups of MCF-7 cells and recorded their migration rates at 6, 24, 48, and 72 h (Figure 3). In the control cells, a line was sliced down the middle of the slide; the migration rates were 0.11 µm/h after 6 h, with a time-dependent increase. The cellular migration was complete at 72 h at the rate of 0.91 µm/h. In the Regorafenib treated group, we applied a 1 μM dose of Regorafenib to the cells, which showed a migration rate of 0.16 µm/h. The migration rate increased in a time-dependent manner but at a slower rate than the control cells. At 72 h, the migration rate was 0.51 µm/h. In the Lenvatinib treated group, we applied a 10 μM dose of Lenvatinib to the cells, which showed non-detectable levels of cell migration at 6 h, and when checked again at 24, 48, and 72 h, it showed migration rates of 0.16, 0.13, and 0.41 µm/h. In the final group, we added a combination of both drugs (1 μM Regorafenib + 10 μM Lenvatinib) to the cells, in order to test their synergistic effect. At 6 h, the migration rates were not detectable. Conversely, the migration rates at 24, 48, and 72 h were 0.06, 0.17, and 0.19 µm/h.

**Figure 4:**
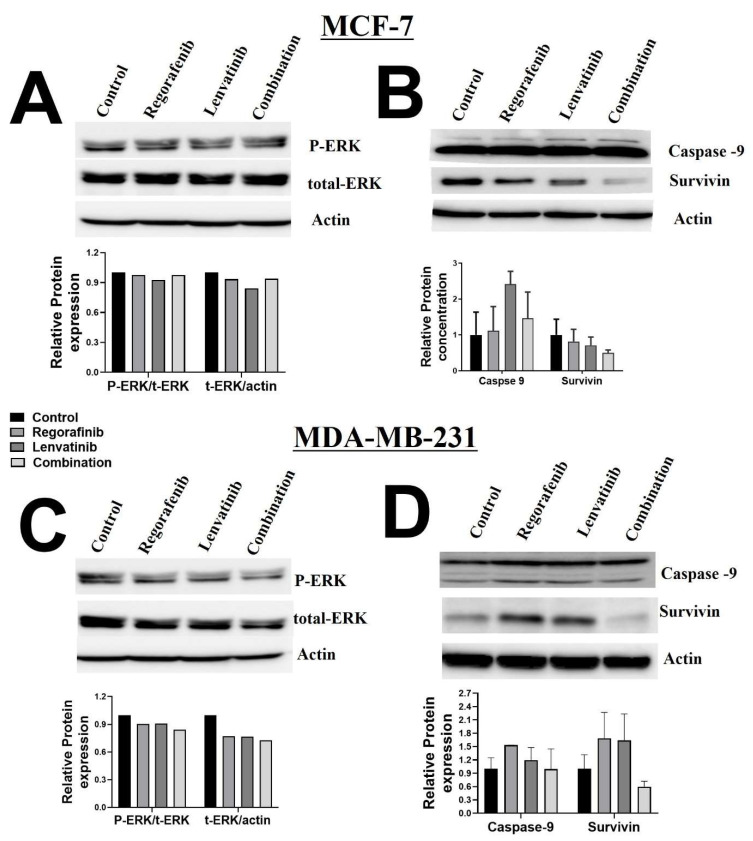
Effect of Regorafenib, Lenvatinib, and combination treatment on protein levels of p-ERK, t-ERK, Capase9, Survivin, and Actin in (**A**,**B**) MCF-7 and (**C**,**D**) MDA-MB-231 (*n* = 1–2/group) cells. Cells were treated with 1 μM Regorafenib for 48 h, 10 μM Lenvatinib for 48 h, and 1 + 10 μM combination for 48 h. Change in protein levels was evaluated using Western blotting.

To get mechanistic insight into the previous findings, we next performed the Western blotting for p-ERK, total-ERK, caspase-9, and Survivin on the MCF-7 and MDA-MB-231 cell lines to measure the expression level of proteins related to cellular proliferation and apoptosis under drug treatment (Figure 4). In MCF-7, there was a significant reduction of survivin expression in drug combination compared to each individual drug (Figure 4B). A similar pattern was shown in MDA-MB-231 cells, but not in the control (Figure 4D). On the other hand, there was no significant change in the expression of caspase, p-ERK, or total-ERK in both cell lines, when treated with Regorafenib, Lenvatinib, or their combination (Figure 4).

**Figure 5:**
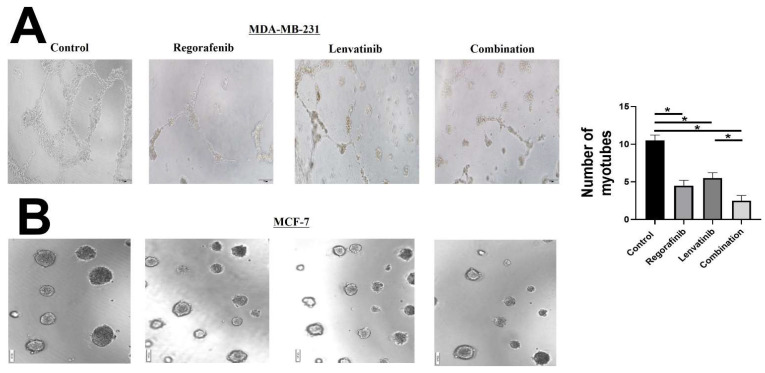
Effect of Regorafenib, Lenvatinib and combination treatment on tube formation assay in the (**A**) MDA-MB-227 and (**B**) MCF-7 cells. There was no tube formation in control group of MCF-7 cells. * Statistically significant, *p*-value < 0.05.

We next performed the tube formation assay, as an established in-vitro assay for angiogenesis, in the MDA-MB-231 and MCF-7 cells in the control and treated groups (Figure 5). We observed the formation of tubes in the control groups of MDA-MB-231 cell lines. We found that each drug significantly reduced the number of tube formations compared to the control group in these cells. Furthermore, the drug combination had a stronger inhibiting effect on tube formation compared to each drug individually. Conversely, there was no tube formation in the control group of MCF-7 cells (data not shown).

**Figure 6:**
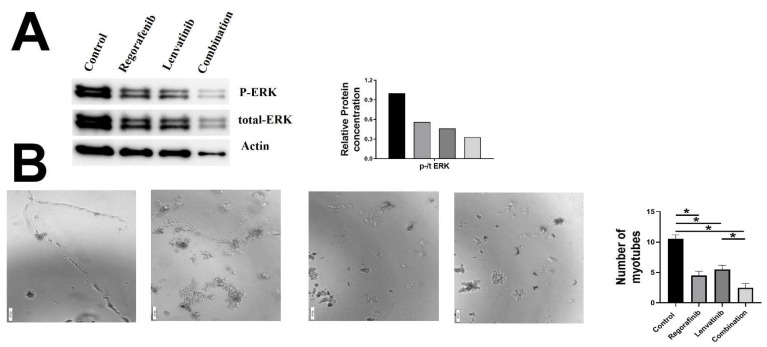
Effect of Regorafenib, Lenvatinib, and combination treatment on (**A**) protein levels of p-ERK, t-ERK, and (**B**) tube formation assay in the HUVEC cells. * Statistically significant, *p*-value < 0.05.

Lastly, we investigated the effects of drugs and their combinations on the HUVEC cell line (Figure 6). We first used Western blot assays to measure the phosphorylation and expression of ERK proteins following the drug administration. A trend towards reduction in ERK phosphorylation was found following drugs treatment when compared to control cells. We next investigated the tube formation assay and found that the drugs treatment led to a reduction in the formation of tubes when compared to control cells.

## 3. Discussion

Both Regorafenib and Lenvatinib have separately shown promising effect against VEGF receptors in various cancer types [13,14,15,16,17,18]. In this study, we have explored the impact of Regorafenib and Lenvatinib, two promising tyrosine kinase inhibitors with anti-angiogenic properties, and their combination against a well-established in-vitro model of breast cancer using MCF-7 and the triple negative MDA-MB-231 cell lines. Our results on the MCF-7 cell line showed that the combination of both drugs had synergistic effects on breast cancer cell survival rates at all variations of drug concentrations, while in MDA-MB-231 cells, the synergistic effect of drug combination occurred at a higher concentration of Lenvatinib. These synergistic effects of drug combinations suggest them as potential therapeutic interventions to treat breast cancer. To our knowledge, no previous study has reported using the combination of these two agents to obtain such an effect on halting the growth and progression of breast cancer cells.

Although not specific to breast cancer, angiogenesis and non-angiogenic vascularization such as vascular mimicry and vascular co-option are vital factors for tumor invasion and progression [5,19,20]. The mechanisms of non-angiogenic vascularization are not completely understood; however, they share similar pathways with angiogenesis such as VEGF-A among others [21]. Therefore, inhibiting angiogenesis and non-angiogenic vascularization through agents targeting the vascular endothelial growth factor carries the potential of increasing the efficacy of chemotherapy. Breast cancer was modeled using MCF-7 and MDA-MB-231 cell lines in the current study. Several studies have shown that the overexpression of VEGF in MCF-7 and MDA-MB-231 cells stimulates cell proliferation in-vivo, and VEGF-A is also known to be the core regulator of angiogenesis in MCF-7 [22]. Therefore, it seems logical that blocking VEGF with anti-VEGFR drugs might be a possible treatment option for breast cancer.

According to our cell viability results, the minimum concentrations of the combination therapy that had a synergistic effect on the MCF-7 cell line were 1 μM of Regorafenib + 10 μM of Lenvatinib. This was also evidenced by the low migration rates (0.02 µm/h), reduced protein expression of survivin, and the high cell death of the cells that were treated with this combination of drugs (98% of MCF-7 cells). In our study, the effect of single treatment with either drug on the cell survival rates was comparable to previously published reports [23,24]. Noteworthy, the apparent reduced effect of these drugs on cell viability in MDA-MB-231 cells and the initial cell proliferation in MCF-7 are statistically non-significant and might be attributed to the initial activation of anti-apoptotic mechanisms. Of note, in our results with MDA-MB-231 cells, each drug alone increased survivin expression while drugs combination reduced its expression. The exact mechanism of this observation is not clear. Survivin has been implicated in tumorigenesis and negative prognosis through various mechanisms such as inhibition of apoptosis, cell cycle, cytokinesis, p53, TGF, and Notch pathways [24]. Whether each drug alone activated any of these mechanisms needs to be further investigated. Furthermore, it seems there is some anti-apoptotic status opposing the effect of those drugs in both breast cancer cell lines we have used. Perhaps because caspase-9 expression is ubiquitous, in particular, in the brain and heart tissues, but not in the breast tissue. Further studies are warranted to clarify this effect and its molecular mechanisms.

Both Lenvatinib and Regorafenib share the same anti-angiogenic effect by inhibiting vascular endothelial growth factor receptors (VEGFRs from 1 to 3) and have been approved for treating different types of tumors. For example, Regorafenib has been used against colorectal carcinoma and hepatocellular carcinoma [25] and Lenvatinib against hepatocellular carcinoma [26]. However, each drug has additional anti-carcinogenic activity. For instance, Regorafenib was shown to have dual-targeted VEGFR2 and TIE2 tyrosine kinase inhibition as anti-angiogenic among other actions, while Lenvatinib blocks other receptors such as fibroblast growth factor receptors (FGFR) 1 to 4, platelet-derived growth factor receptor (PDGFR) alpha, RET, and KIT [26]. Thus, reducing the tube formation capacity that was evidenced in our MDA-MB-231 cells by targeting two independent molecular pathways may be an effective anti-cancer strategy.

In addition to their anti-angiogenic activities, both Regorafenib and Lenvatinib demonstrate their potency in combination with other anti-cancer therapies. For instance, Regorafenib is shown to sensitize triple negative breast cancer cells to radiation therapy [27], while the combination of Lenvatinib with the immune check point inhibitor pembrolizumab demonstrates promising activity against a number of solid tumors including breast, endometrial and renal cancer [28,29].

Triple negative breast cancer TNBC is known to present with a more aggressive dis-ease pattern compared to receptor-positive breast cancer and has a higher probability of distant metastases [30,31,32,33]. It has been also shown to have an association with the clinical indicative factor of aggressive metastatic disease [32,33]. Consistent with this, patients with metastatic TNBC show poor disease outcomes [34,35]. Further targeting the SHP1 signaling pathway with Regorafenib demonstrates a promising potential therapy for TNBC [36]. Regorafenib has been also shown to be useful in inhibiting migration and proliferation in some subtypes of breast cancer cells [37].

In addition to targeting VEGF, novel approaches such as targeting the P2X7 receptors and using microRNAs could serve as a potential therapy for breast cancer in the future [18,38].

The main limitation of the current study is that it does not recapitulate complex physiological interactions among multiple systems as in an in-vivo model. However, since all the MCF-7, MDA-MB-231, and HUVEC cell lines are well-characterized in myriad studies that have used them [20,22,26,39], extrapolating our study of the effect of Regorafenib and Lenvatinib on breast cancer growth and progression into an in-vivo model seems reasonable to be considered. Ultimately, clinical trials will be required to validate the beneficial combined effect of Regorafenib and Lenvatinib in patients with breast cancer of various molecular subtypes.

## 4. Material and Methods

### 4.1. Cell Culture

MCF-7 (ATCC^®^ HTB-22™), MDA-MB-231 (ATCC^®^ HTB-26™) human breast cancer cells, were purchased from ATCC^®^, and human umbilical vascular endothelial cells (HUVECs, ScienCell Research Laboratories, Carlsbad, CA, USA). Dulbecco’s Modified Eagle’s Medium (DMEM) was used to maintain the cells at 37 °C and 5% CO_2_ as it was supplemented with 4 mM glutamine, 2 μg/mL insulin, 10% fetal calf serum, 1 mM of sodium pyruvate, 1 mM of nonessential amino acids, and penicillin/streptomycin antibiotics [40,41]. HUVEC cells were grown in EGM-2 supplemented with 10% FBS [39]. Cells were maintained at 37 °C in a humidified incubator containing 5% CO_2_. The passage number of all cells was between 3 and 5. Regorafenib (Catalog No.: A10250) and Lenvatinib (Catalog No.: A10340) were purchased from AdooQ^®^ Bioscience as a dissolved solution of 10 mM in 1 mL DMSO.

### 4.2. Cell Viability Assay

Cell viability was determined in cells treated with the drugs by using a colorimetric assay MTT (3-(4,5-dimethylthiazol-2-yl)-2,5-diphenyltetrazolium bromide (Sigma-Aldrich, St. Louis, MO, USA). Cells were seeded in 96-well plates at a density of 4 × 10^3^ cells per well and then incubated overnight to achieve a confluency of 60%. Afterward, the cells were divided into three groups; two of the cell groups were treated with various concentrations of each drug alone, Regorafenib at 1 µM, 5 µM, 10 µM, and Lenvatinib at 10 µM, 50 µM, 100 µM, and the third group was treated with a combination of both drugs as shown in Figure 1 and Figure 2. Cells were cultured for 24, 48, and 72 h. After that MTT salt was added and mixed with the cells then kept for 2 h incubation in a humidified incubator at 37 °C and 5% CO_2_. The product of MTT formazan was dissolved in DMSO and then a reading of absorbance was taken at 570 nm using a microplate reader [42].

### 4.3. Cell Migration Assay

Cancerous cells migrate through various complex processes [19]. A wound healing assay is a standard method to investigate cell migration. We used this assay to evaluate the migration of both MCF-7 and MDA-MB-231 breast cancer cells that were treated in Regorafenib 1 µM, Lenvatinib 10 µM, and the combination of both agents. Then, 1.5 × 10^6^ were plated in 3 mL of media in 6 well plates. The cells were wounded in the middle of each well by scratching the culture plate with a pipette tip. The debris was then washed with PBS before adding the media and the treatment. The next step was treating the plates with Regorafenib 1 µM alone, Lenvatinib 10 µM alone, and a combination of both drugs. We used a control that contained the cells and the medium only. Quantitative analysis of the cell migration was performed using Image J software, National Institutes of Health (NIH), USA (http://rsb.info.nih.gov/ij/index.html (accessed on 12 September 2021). The migration rate was calculated using the following equation:

Migration rate = (mean width at 24 h − mean width at 48 h)/mean width at 24 h.

### 4.4. Western Blot

Cells were lysed in ice-cold NP-40 lysis buffer (1.0% NP-40, 150 mM of NaCl, 50 mM of Trs-Cl, pH 8.0) containing protease inhibitor cocktail tablets (Cat. No. S8830; Sigma, Taufkirchen, Germany). The protein concentration of cell lysate was quantified using the standard Bradford method (Cat. No. 500-0006; Bio-Rad, Hercules, CA, USA). Then, 50 μg of lysate protein aliquots were separated by 12% sodium dodecyl sulfate–polyacrylamide gel electrophoresis (SDS-PAGE) and transferred onto a nitrocellulose membrane (Bio-Rad, Hercules, CA, USA). Next, 5% skimmed milk powder was used to block the membrane at room temperature for 1 h. The membrane was then washed with TBST and incubated with the following primary antibodies (caspae-9 [cat# 9508], survivin [cat# 2808], p-ERK/ERK [cat# 9926], and β-actin [cat# 3700], all from Cell Signaling Technology, Danvers, MA, USA) overnight at 4 °C. Secondary antibodies (Cell Signaling Technology, Danvers, MA, USA) were incubated with the membrane at 1:1000 dilution for 1 h at room temperature. Chemiluminescence was detected using the ECL kit (Thermo Scientific Pierce, Waltham, MA, USA). Bio-Rad Image Lab software (ChemiDoc™ Touch Gel and Western Blot Imaging System; Bio-Rad) was used to detect and quantify protein bands. Protein levels were normalized to β-actin and ratios were calculated based on the values of control (untreated) samples [43].

Total protein was extracted by adding 50 μL NP40 to each tube, and then incubated for 45 min in ice and vortexed every 10 min. Protein concentration was evaluated by a cuvette spectrophotometer. The protein sample was separated by sodium dodecyl sulfate–polyacrylamide gel electrophoresis (SDS-PAGE) and then transferred to nitrocellulose (NC) membranes by the semi-dry blotting method. The membranes were blocked in 5% skim milk at room temperature for one hour over the shaker. Then, the membranes were incubated with primary antibodies against, caspase-9, survivin, p-ERK, ERK, and β-actin (1:1000 dilution) at 4 °C overnight. On the next day, the membranes were incubated with matched secondary antibodies (1:800 dilutions) at room temperature for 45 min. Finally, protein bands were visualized by a chemiluminescence system. The above antibodies were purchased from Cell Signaling Technology, Danvers, MA, USA.

### 4.5. Tube Formation Assay

The assay was performed with the tube formation assay kit (Abcam, Cambridge, UK). Briefly, according to the manufacturer protocol 50 µL/well of the extracellular matrix solution (ECM) was added to a 96 well culture plate and incubated for 1 h at 37 °C to allow gel formation. Afterward, 2 × 10^4^ cells per 100 µL were seeded in each well, followed by being left untreated or treated with Regorafenib 1 µM, Lenvatinib 10 µM, and the combination of both agents for 72 h. Several images were captured by a phase-contrast inverted microscope at 10× magnification [44].

### 4.6. Data Analysis

Mean ± standard error of the mean (SEM) was calculated for the sample’s replicates in each group. One-way ANOVA was used to compare the difference among groups. A *p*-value < 0.05 was considered significant. Compusyn software was used to calculate the combination index to detect the drug–drug interaction (CI < 1 denotes synergy, CI > 1 denotes antagonism, whereas CI = 1 denotes addition) [45].

## 5. Conclusions

This in-vitro study reveals the synergistic anti-proliferative and anti-angiogenic potential of the combination therapy of Lenvatinib and Regorafenib, inhibiting the progression of breast cancer at the molecular level. The dual anti-angiogenic effect of both drugs shows a better effect than each drug alone. Furthermore, this synergistic effect was evidenced by a marked decrease in tumor markers in all conducted assays. Although both drugs have been approved in treating different types of cancer, to our knowledge, this is the first study to report the synergistic effect of their combination on breast cancer cell lines (MCF-7 and MDA-MB-231). Due to the potential usefulness of this therapeutic approach for breast cancer treatment, further in-vivo and detailed studies are warranted in the future.

## Data Availability

Not applicable.

## Supplementary Materials

The following supporting information can be downloaded at: https://www.mdpi.com/article/10.3390/ijms23084408/s1.
