# Correction: Bajbouj et al. Synergistic Anti-Angiogenic Effect of Combined VEGFR Kinase Inhibitors, Lenvatinib, and Regorafenib: A Therapeutic Potential for Breast Cancer. *Int. J. Mol. Sci.* 2022, *23*, 4408

**DOI:** 10.3390/ijms27020743

**Published:** 2026-01-12

**Authors:** Khuloud Bajbouj, Rizwan Qaisar, Mohammed A. Alshura, Zeinab Ibrahim, Mohamad B. Alebaji, Amenah W. Al Ani, Hanadi M. Janajrah, Mariah M. Bilalaga, Abdelrahman I. Omara, Rebal S. Abou Assaleh, Maha M. Saber-Ayad, Adel B. Elmoselhi

**Affiliations:** College of Medicine, University of Sharjah, Sharjah P.O. Box 27272, United Arab Emiratesrqaisar@sharjah.ac.ae (R.Q.);

Figure 3A has been updated to replace an inadvertent duplication from Figure 3B. In the original publication [1], the panel showing MCF-7 cells treated with Regorafenib for 24 h in the wound-healing assay was incorrectly duplicated due to a mix-up error. The corrected panel now shows the appropriate image for the Regorafenib (24 h) treatment. The authors state that the scientific conclusions are unaffected. This correction was approved by the Academic Editor. The original publication has also been updated.

**Figure 3:**
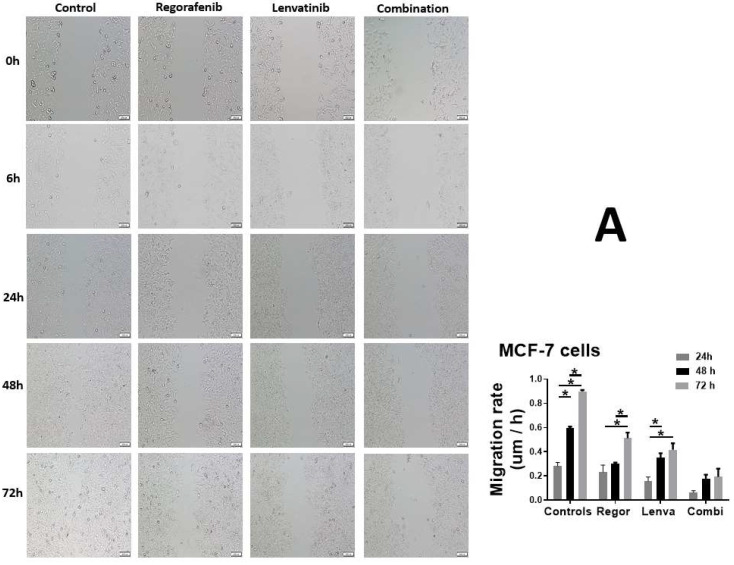

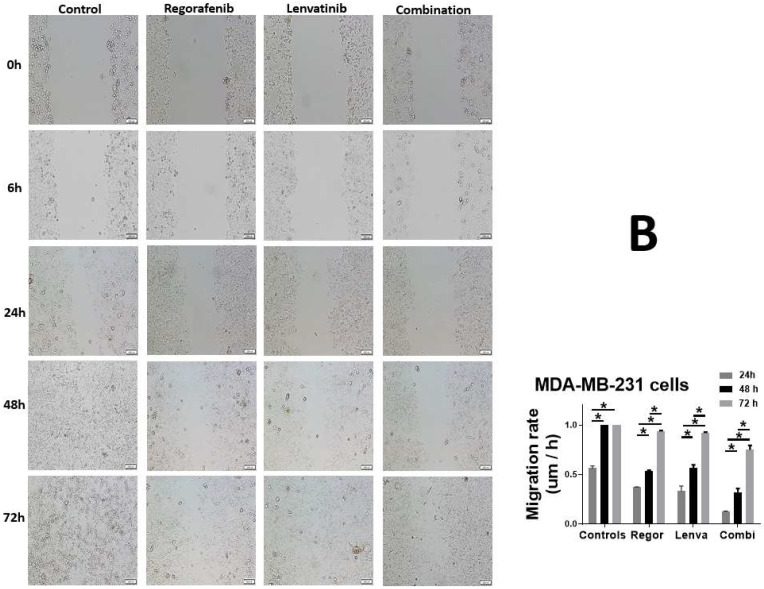
Effect of Regorafenib and Lenvatinib on cell migration. A scratch test was used to assess (**A**) MCF-7 and (**B**) MDMBA cell migration, and the width of the scratch was noted at the pre-defined time points of 6, 24, and 48 h. The scale bar corresponds to 100 µm for all panels. The untreated control cells showed significant cellular migration, while treatment with Regorafenib and Lenvatinib, either alone or in combination, prevented the migration of MCF-7 and MDMBA cells, confirming the anti-metastatic potential of these drugs. * Statistically significant from the control cells, *p*-value < 0.05.
