# Supplementary material for: Synergistic Anti-Angiogenic Effect of Combined VEGFR Kinase Inhibitors, Lenvatinib, and Regorafenib: A Therapeutic Potential for Breast Cancer
Source: Int J Mol Sci. 2022 Apr 16;23(8):4408. doi: 10.3390/ijms23084408 (PMC9028329; doi:10.3390/ijms23084408)
Supplement: Supplementary file 1 [file ijms-23-04408-s001.zip › ijms-1629892-supplementary.pdf]

Supplementary figures

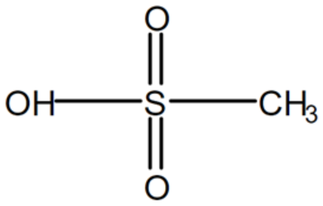

CAS Name : Lenvatinib mesylate,
Molecular Formula : C21H19ClN4O4.CH4O3S
CAS Registry Number® 857890-39-2
Reference :
Lenvatinib mesylate. *CAS Common Chemistry*. CAS, a division of the American Chemical Society, n.d. https://commonchemistry.cas.org/detail?ref=857890-39-2 (retrieved 2021-05-05) (CAS RN: 857890-39-2). Licensed under the Attribution-Noncommercial 4.0 International License (CC BY-NC 4.0).

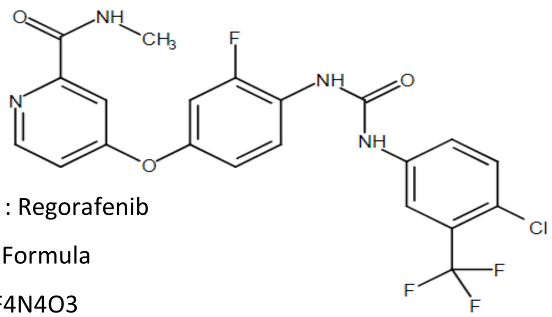

CAS Name : Regorafenib

Molecular Formula

C21H15ClF4N4O3

CAS Registry Number®

755037-03-7

Reference :

Regorafenib. CAS Common Chemistry. CAS, a division of the American Chemical Society, n.d. https://commonchemistry.cas.org/detail?cas_rn=755037-03-7 (retrieved 2021-05-05) (CAS RN: 755037-03-7).

Supplementary Figure S1: Chemical structure of Lenvatinib and Regorafenib

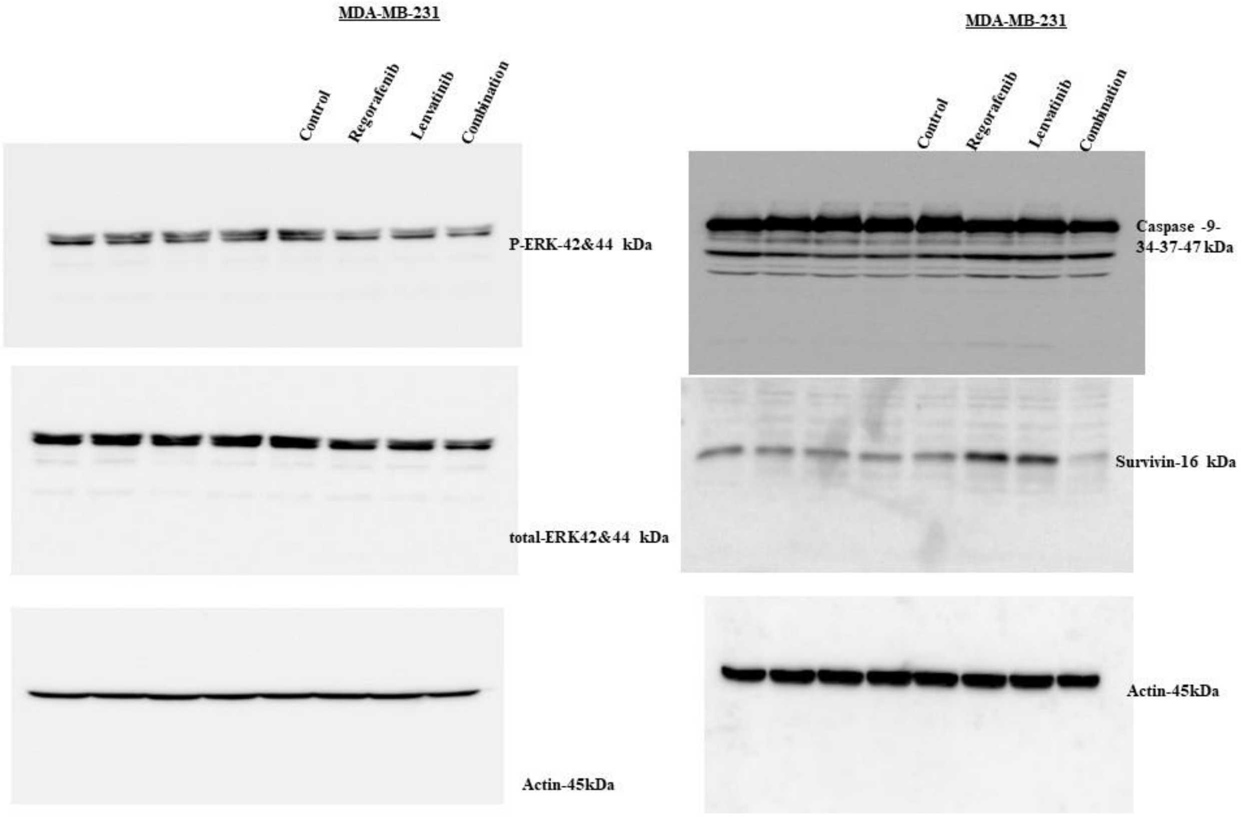

Supplementary Figure S2: Original western blot for caspase, surviving, P-ERK and total-ERK.

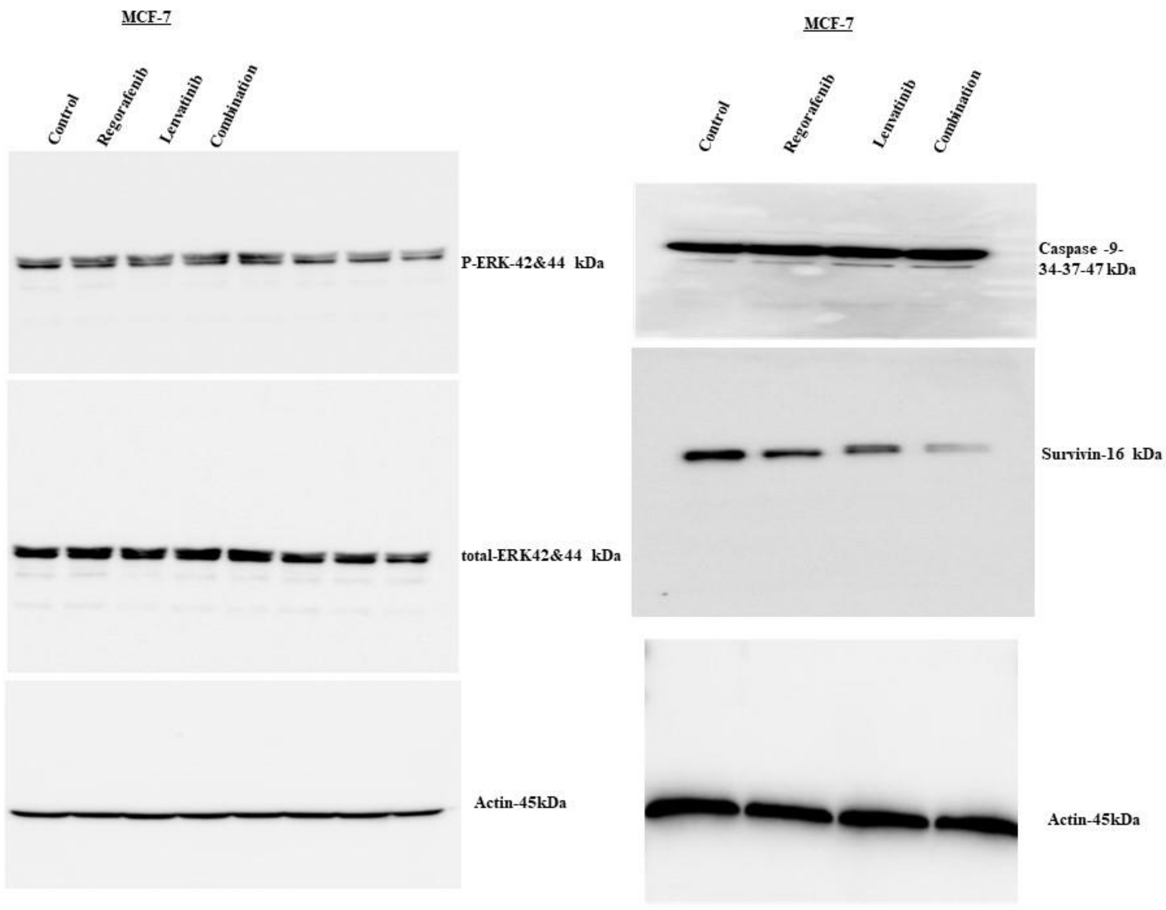

Supplementary Figure S3: Original western blot for caspase, surviving, P-ERK and total-ERK.

Supplementary CompuSyn Report 1-MCF7 cells

Experiment Name: REG-LEN
Date: 9.5.2021
File Name: C:\Users\USER\Downloads\com72_MCF7 EXP.cse
Description

Drug: REG (REG) [uM]
Drug: LEN (LEN) [uM]
Drug Combo: Com 72 (Com72) (REG+LEN)

Data for Drug: REG [uM]

Dose	Effect
1.0	0.99
5.0	0.9
10.0	0.87

3 data points entered.

X-int: 1.59249
Y-int: 1.95302 +/- 0.17272
m: -1.2264 +/- 0.24520
Dm: 39.1282
r: -0.9806

Data for Drug: LEN [uM]

Dose	Effect
10.0	0.99
50.0	0.64
100.0	0.64

3 data points entered.

X-int: 2.00920
Y-int: 3.77364 +/- 0.93455
m: -1.8782 +/- 0.57640
Dm: 102.140
r: -0.9560

Data for Non-Constant Combo: Com72 (REG+LEN)

Dose REG	Dose LEN	Effect
5.0	10.0	0.87
5.0	50.0	0.62
5.0	100.0	0.47

Dose REG	Dose LEN	Effect
10.0	10.0	0.53
10.0	50.0	0.48
10.0	100.0	0.4

6 data points entered.

Dose-Effect Curve

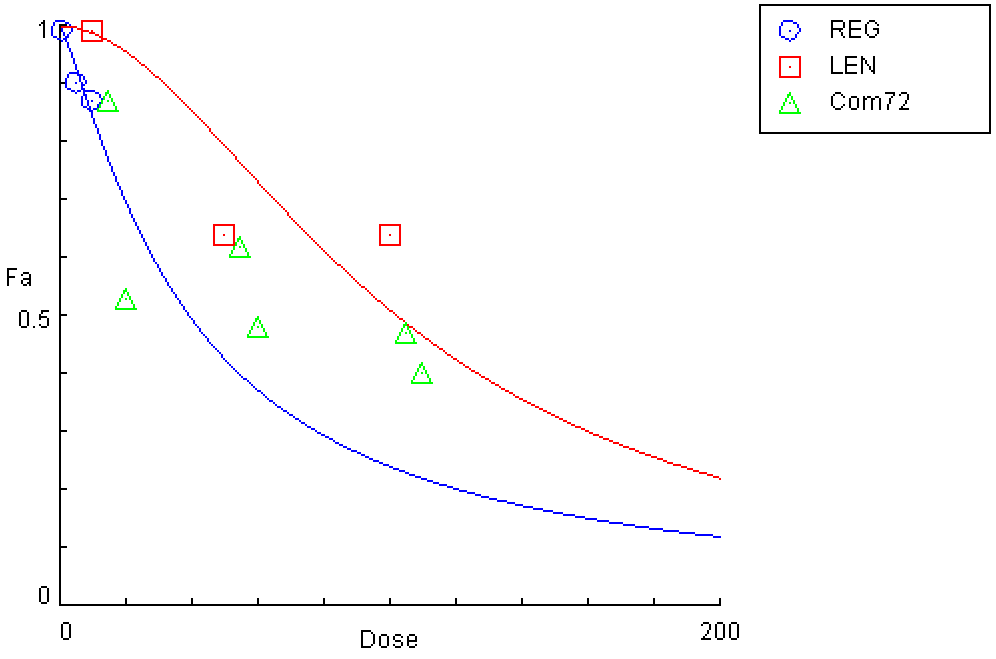

Median-Effect Plot

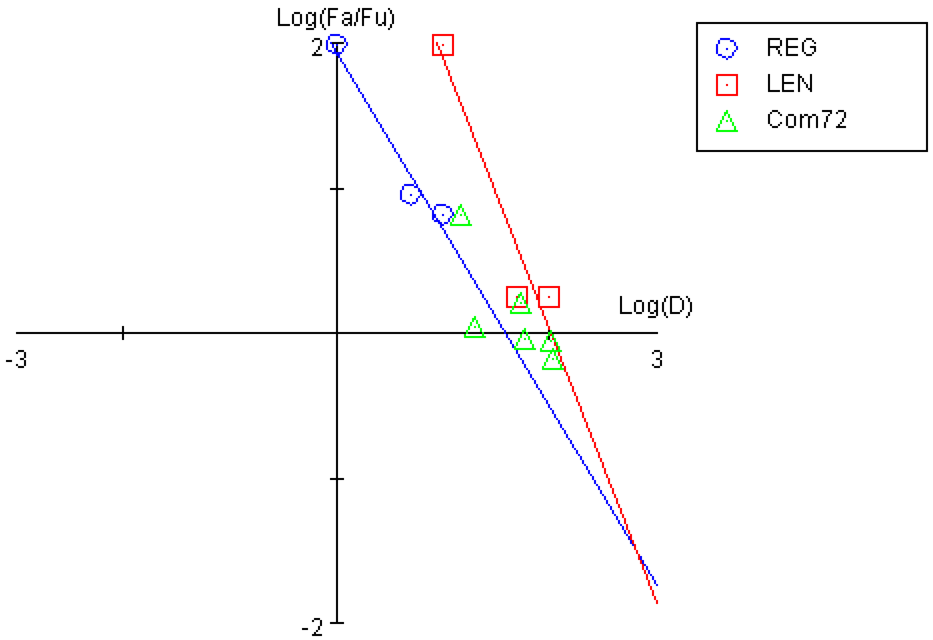

CI Data for Non-Constant Combo: Com72 (REG+LEN)

**Dose REG**	**Dose LEN**	**Effect**	**CI**
5.0	10.0	0.87	0.87146
5.0	50.0	0.62	0.82577
5.0	100.0	0.47	1.03424
10.0	10.0	0.53	0.38625
10.0	50.0	0.48	0.70852
10.0	100.0	0.4	0.97257

Combination Index Plot

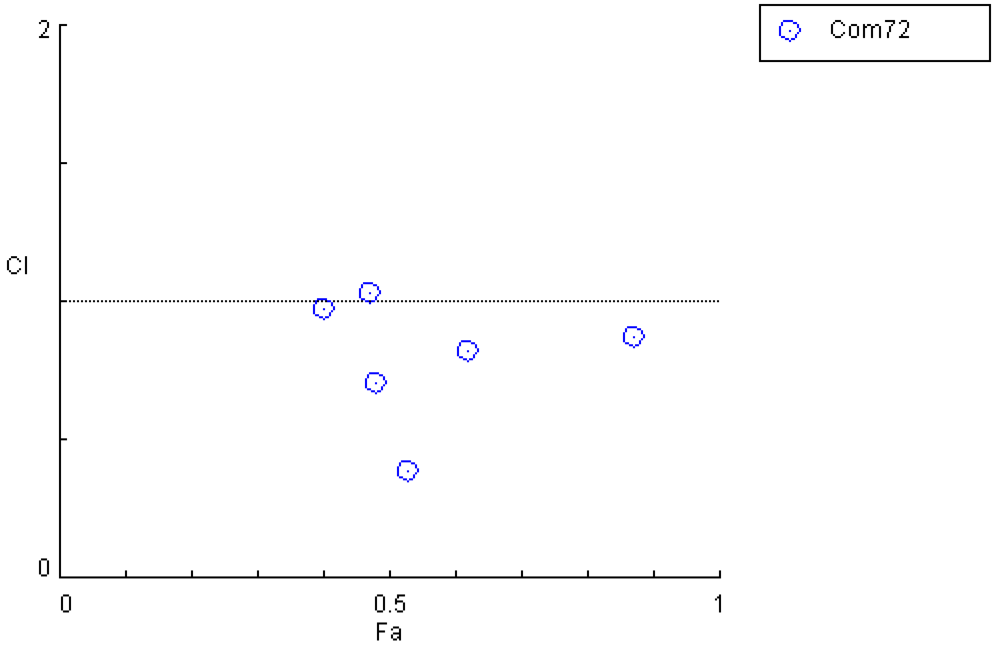

DRI Data for Non-Constant Combo: Com72 (REG+LEN)

Fa	Dose REG	Dose LEN	DRI REG	DRI LEN
0.87	8.30456	37.1224	1.66091	3.71224
0.62	26.2500	78.7041	5.25001	1.57408
0.47	43.1555	108.887	8.63110	1.08887
0.53	35.4768	95.8108	3.54768	9.58108
0.48	41.7672	106.587	4.17672	2.13174
0.4	54.4596	126.751	5.44596	1.26751

DRI Plot for Non-Constant Combo: Com72 (REG+LEN)

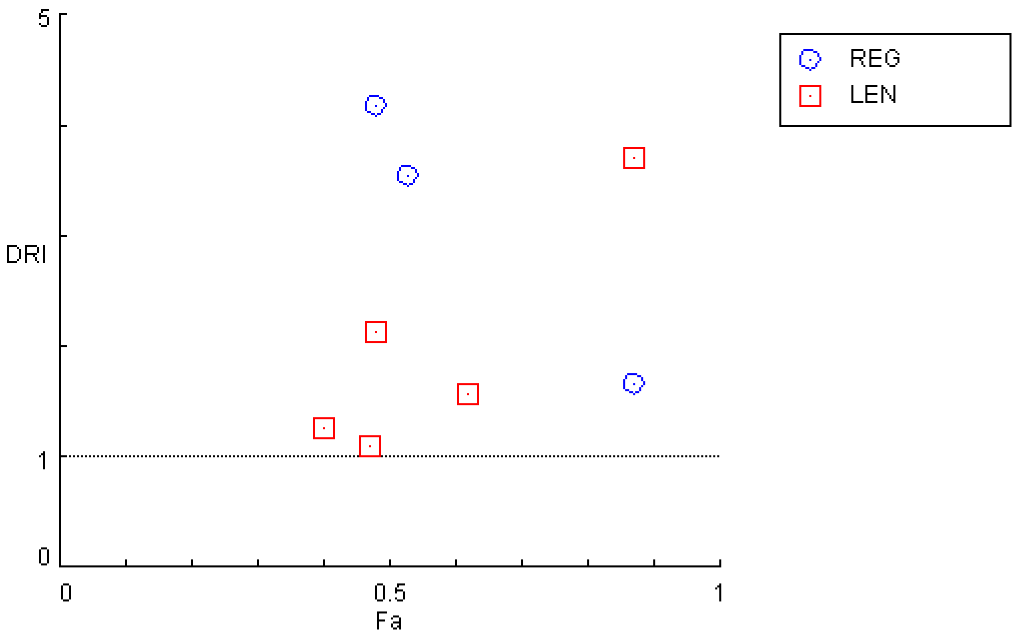

Normalized Isobologram for Combo: Com72 (REG+LEN)

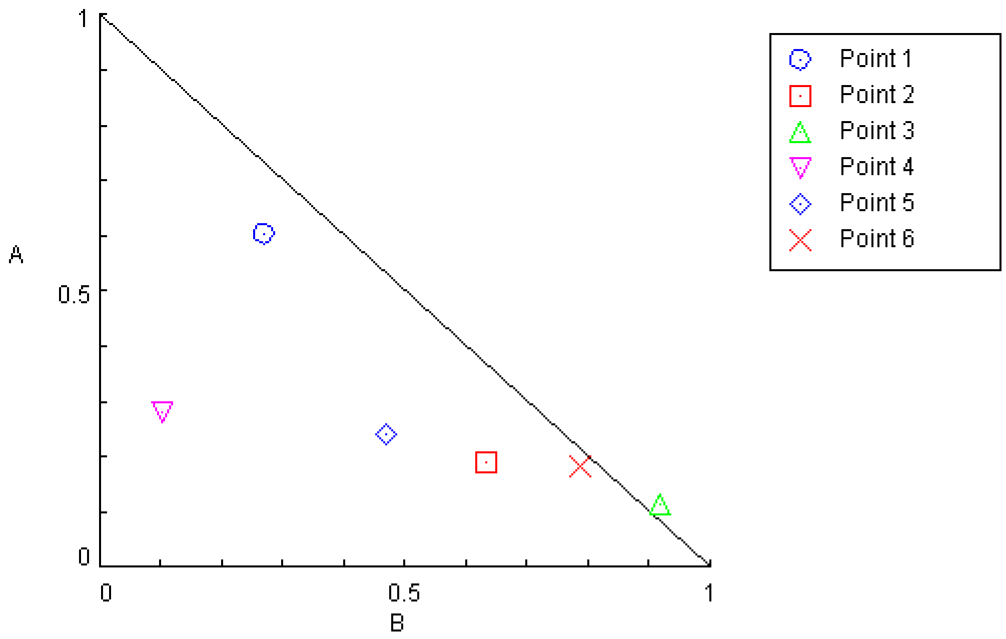

Summary Table

Experiment Name: REG-LEN

Date: 9.5.2021

File Name: C:\Users\USER\Downloads\com72_MCF7 EXP.cse

Description

Drug: REG (REG) [uM]

Drug: LEN (LEN) [uM]

Drug Combo: Com 72 (Com72) (REG+LEN)

Drug/Combo	Dm	m	r
REG	39.1282	-1.2264	-0.9806
LEN	102.140	-1.8782	-0.9560

CI values at:

Combo	ED50	ED75	ED90	ED95

Data for Fa = 0.5

Drug/Combo	CI value	Dose REG	Dose LEN
REG		39.1282	
LEN			102.140

Data for Fa = 0.75

Drug/Combo	CI value	Dose REG	Dose LEN
REG		15.9752	
LEN			56.9066

Data for Fa = 0.9

Drug/Combo	CI value	Dose REG	Dose LEN
REG		6.52232	
LEN			31.7051

Data for Fa = 0.95

Drug/Combo	CI value	Dose REG	Dose LEN
REG		3.54647	
LEN			21.2986

Data for Fa = 0.97

Drug/Combo	CI value	Dose REG	Dose LEN
REG		2.29891	
LEN			16.0478

Supplementary CompuSyn Report 2- MD-NB 231 cells

Experiment Name: REG_LEN_72_TN
Date: 9.5.2021
File Name: C:\Users\USER\Downloads\com72_TN.cse
Description

Drug: REG (REG) [uM]
Drug: LEN (LEN) [uM]
Drug Combo: Com72_TN (TN72) (REG+LEN)

Data for Drug: REG [uM]

Dose	Effect
1.0	0.99
5.0	0.96
10.0	0.91

3 data points entered.

X-int: 2.06953
Y-int: 2.01034 +/- 0.05960
m: -0.9714 +/- 0.08461
Dm: 117.364
r: -0.9962

Data for Drug: LEN [uM]

Dose	Effect
10.0	0.99
50.0	0.86
100.0	0.92

3 data points entered.

X-int: 2.75905
Y-int: 2.96456 +/- 0.98479
m: -1.0745 +/- 0.60738
Dm: 574.186
r: -0.8705

Data for Non-Constant Combo: TN72 (REG+LEN)

Dose REG	Dose LEN	Effect
5.0	10.0	0.99
5.0	50.0	0.97

Dose REG	Dose LEN	Effect
5.0	100.0	0.74
10.0	100.0	0.7

4 data points entered.

Dose-Effect Curve

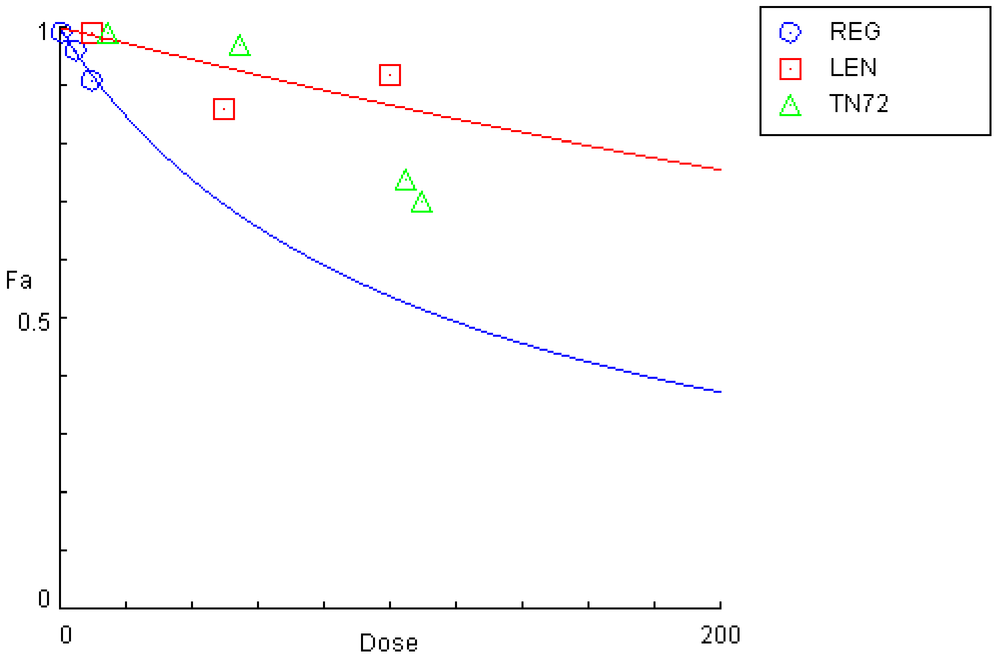

Median-Effect Plot

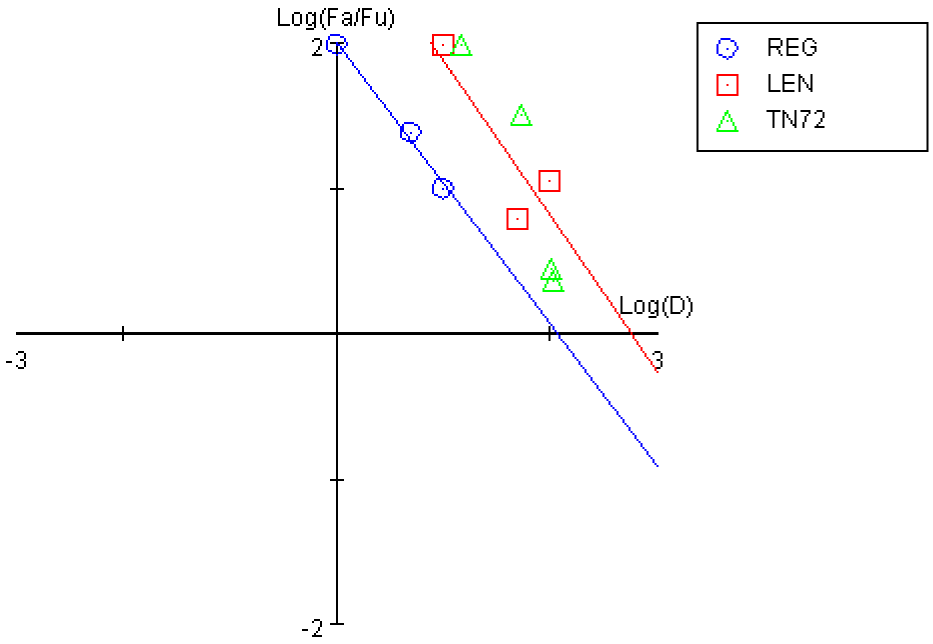

CI Data for Non-Constant Combo: TN72 (REG+LEN)

Dose REG	Dose LEN	Effect	CI
5.0	10.0	0.99	6.08257
5.0	50.0	0.97	3.73862
5.0	100.0	0.74	0.58606
10.0	100.0	0.7	0.58703

Combination Index Plot

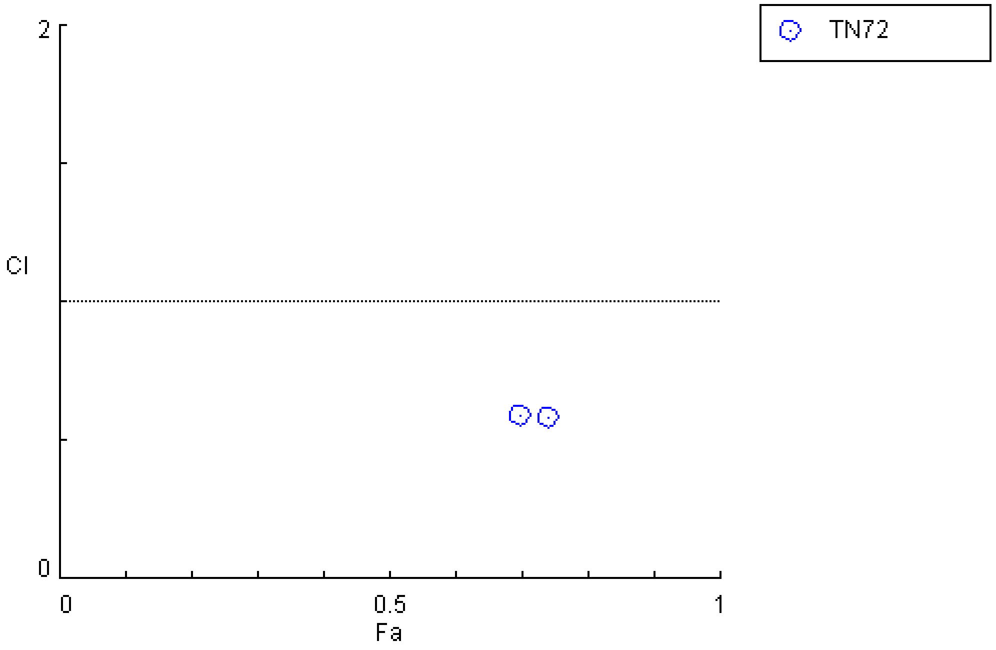

DRI Data for Non-Constant Combo: TN72 (REG+LEN)

Fa	Dose REG	Dose LEN	DRI REG	DRI LEN
0.99	1.03547	7.97545	0.20709	0.79754
0.97	3.27667	22.5970	0.65533	0.45194
0.74	39.9853	216.912	7.99706	2.16912
0.7	49.0594	260.966	4.90594	2.60966

DRI Plot for Non-Constant Combo: TN72 (REG+LEN)

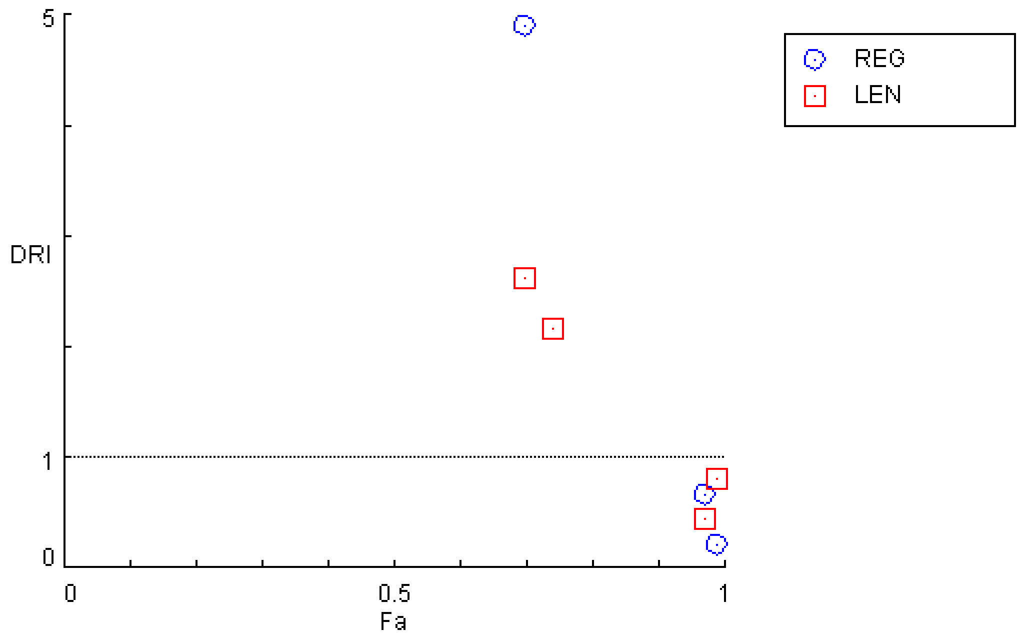

Normalized Isobologram for Combo: TN72 (REG+LEN)

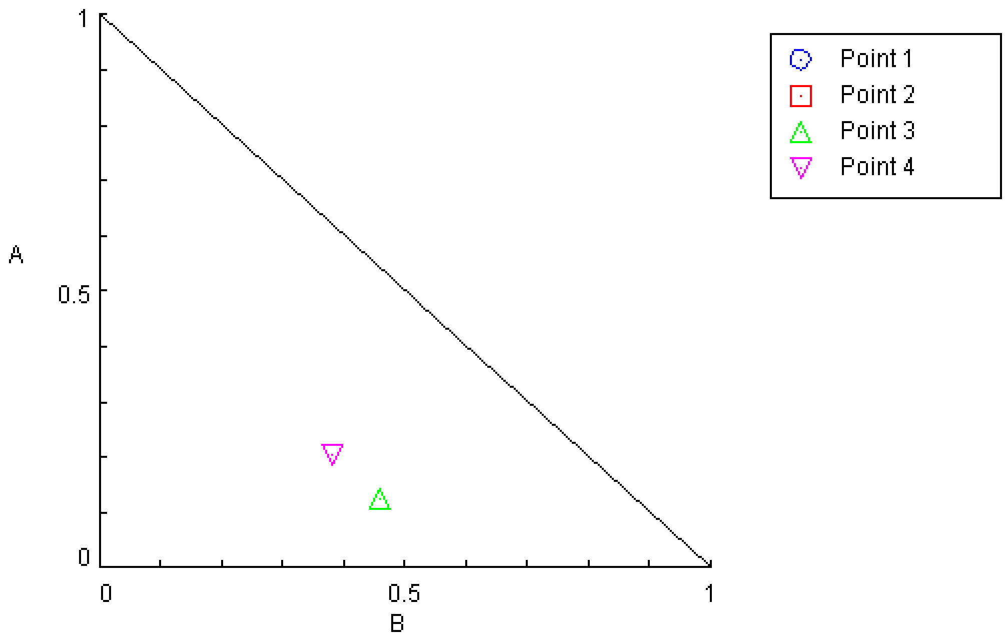

Summary Table

Experiment Name: REG_LEN_72_TN

Date: 9.5.2021

File Name: C:\Users\USER\Downloads\com72_TN.cse
Description

Drug: REG (REG) [uM]
Drug: LEN (LEN) [uM]
Drug Combo: Com72_TN (TN72) (REG+LEN)

Drug/Combo	Dm	m	r
REG	117.364	-0.9714	-0.9962
LEN	574.186	-1.0745	-0.8705

CI values at:

Combo ED50 ED75 ED90 ED95

Data for Fa = 0.5

Drug/Combo	CI value	Dose REG	Dose LEN
REG		117.364	
LEN			574.186

Data for Fa = 0.75

Drug/Combo	CI value	Dose REG	Dose LEN
REG		37.8760	
LEN			206.541

Data for Fa = 0.9

Drug/Combo	CI value	Dose REG	Dose LEN
REG		12.2235	
LEN			74.2948

Data for Fa = 0.95

Drug/Combo	CI value	Dose REG	Dose LEN
REG		5.66406	
LEN			37.0632

Data for Fa = 0.97

Drug/Combo	CI value	Dose REG	Dose LEN
REG		3.27667	
LEN			22.5970
